# Assessing the Effect of Cytoreduction on Solitary, Resectable Lesions in Primary Central Nervous System Lymphoma

**DOI:** 10.3390/cancers17060917

**Published:** 2025-03-07

**Authors:** Chaejin Lee, Yukyeng Byeon, Gung Ju Kim, Juhee Jeon, Chang Ki Hong, Jeong Hoon Kim, Young-Hoon Kim, Young Hyun Cho, Seok Ho Hong, Sang Joon Chong, Sang Woo Song

**Affiliations:** 1 Department of Neurosurgery, School of Medicine, Kyungpook National University, Daegu 41944, Republic of Korea; cjleee01@gmail.com; 2 Department of Neurological Surgery, Asan Medical Center, University of Ulsan College of Medicine, Seoul 05505, Republic of Korea

**Keywords:** primary central nervous system lymphoma, resection, remission, cytoreduction, clinical course

## Abstract

**Background/Objectives:** The management of primary central nervous system lymphoma (PCNSL) has traditionally prioritized diagnostic biopsy, with surgical resection often considered secondary due to risks and potential bias in previous studies, which included patients with deep or multiple tumors. This study aims to evaluate the impact of surgical resection on survival in patients with solitary, resectable PCNSL. **Methods:** We conducted a retrospective analysis of PCNSL patients treated via brain biopsy or surgical resection at our institution between January 2010 and December 2022. Cases with deep-located tumors (corpus callosum, basal ganglia, thalamus, and brainstem) or multiple lesions were excluded. Survival and clinical outcomes were compared between the two groups. **Results:** A total of 79 patients (30 resection and 49 biopsy) were included. No significant differences were observed between groups regarding demographics, comorbidities, tumor characteristics, or International Extranodal Lymphoma Study Group scores. Preoperative midline shifting (*p* = 0.048) and steroid use (*p* < 0.001) were higher in the resection group, which also demonstrated greater symptom improvement (*p* < 0.001). The complication rates were comparable between groups. The 5-year overall survival (OS) was 81.3% (resection) vs. 80.1% (biopsy), and the 5-year progression-free survival (PFS) was 53.6% (resection) vs. 60.3% (biopsy), with no significant differences in OS or PFS by Cox regression analysis. **Conclusions:** Surgical resection does not improve OS or PFS in solitary, resectable PCNSL, though it may provide symptomatic relief in select cases. Further prospective studies are needed to define its role in PCNSL management.

## Simple Summary

Previous studies on the surgical role in primary central nervous system lymphoma (PCNSL) have been biased, including patients with deep or multiple tumors unsuitable for resection. To address this, we focused exclusively on patients with solitary, resectable lesions to evaluate the true impact of surgical resection on survival. Our findings aim to clarify the potential benefits of resection in carefully selected PCNSL cases, providing a new perspective on its role in treatment.

## 1. Introduction

Primary central nervous system lymphoma (PCNSL) is a rare and aggressive tumor, representing approximately 2.4–3% of primary central nervous system (CNS) tumors [1,2]. Although there have been advancements in PCNSL treatment over the past 40 years, a definitive gold standard for therapy remains elusive [3,4,5]. The role of resection is largely confined to histological confirmation, as PCNSL is a systemic hematologic malignancy rather than a focal surgical disease. Furthermore, PCNSL stands out as a highly chemosensitive tumor compared to other primary CNS tumors, such as glioblastoma multiforme and meningioma, making chemotherapy (CTx) the cornerstone of treatment [6,7]. However, the evidence supporting cytoreduction predominantly stems from small-scale retrospective studies lacking prospective validation. Consequently, the actual impact of cytoreduction through surgery on subsequent prognosis remains uncertain.

Diagnostic discrepancies can occur prior to the histological confirmation of PCNSL. Due to nonspecific symptoms and potential misinterpretation on imaging studies, PCNSL can be mistaken for other tumors or infections, leading to cases where gross total resection (GTR) is considered before a definitive diagnosis. This raises questions about the prognosis compared to cases where only a biopsy is performed.

Investigating effective treatment approaches is crucial, especially for solitary, resectable lesions. GTR could be a promising intervention in these cases, primarily owing to the potential for immediate postoperative complete response (CR), which can lead to symptom improvement and influence the disease’s clinical course.

This study was motivated by the question of whether cytoreduction, when feasible in cases of solitary, resectable PCNSL, could offer a beneficial effect. Additionally, to address this question, we conducted a retrospective comparative analysis of a patient cohort diagnosed with PCNSL following brain biopsy or resection. This study will offer an overview of the existing literature, highlighting gaps in our understanding of surgery’s impact on the disease’s clinical course. Ultimately, this study aimed to provide insights that can contribute to a better understanding of ideal conditions and factors to consider for surgical interventions in PCNSL, especially when resection is feasible.

## 2. Materials and Methods

### 2.1. Patient Selection and Data Collection

We conducted a retrospective study at Asan Medical Center from January 2010 to December 2022 on patients diagnosed with PCNSL via resection or brain biopsy. Patients with multifocal or multiple lesions were excluded due to challenges in achieving GTR [8]. Secondary lymphoma cases were excluded due to their common leptomeningeal spread and poorer prognosis compared to PCNSL [9]. Patients who were lost to follow-up before completing the first chemotherapy (CTx) cycle were excluded because the immediate radiologic response after the first CTx, one of the key outcome measures, could not be assessed. For the definition of “deep location”, unresectable locations encompassing the corpus callosum, basal ganglia, thalamus, and brainstem were considered, and such cases were excluded from the research. In this study, deep location differed from the cerebellum-inclusive definition in the International Extranodal Lymphoma Study Group (IELSG) score [10], and solitary tumors in the cerebellum were included in this study cohort. Tumors located outside the deep locations were considered “resectable”.

Patients were categorized into two groups: resection and biopsy. The resection group included cases with confirmed GTR radiologically and intra-operatively, whereas the biopsy group comprised cases where only a small amount of tissue was collected for pathological diagnosis. Biopsy techniques included stereotactic biopsy utilizing the Leksell stereotactic frame (Elekta AB, Stockholm, Sweden), navigation-guided needle biopsy, and open biopsy with a small craniotomy. Clinical data, including sex, age, comorbidities, Karnofsky performance status (KPS) score, IELSG score, preoperative steroid use within 3 days, and initial symptom presentation, were collected. The KPS score was evaluated before surgery and one month postoperatively. Radiological data included tumor location, diameter, peritumoral edema, and midline shifting. Tumor location was determined by identifying the lobe of the tumor epicenter. The anatomical sites were categorized by distinguishing lobes within the cerebrum. Distinctions were made for other locations, including the cerebellum, 4th ventricle, or suprasellar region. Tumor diameter was measured in the axial, coronal, and sagittal planes, selecting the section that showed the maximal diameter. Furthermore, midline shifting was assessed using a 5 mm criterion. Postoperative complications were examined, encompassing regional complications such as surgical site infection and postoperative bleeding, systemic complications such as pneumonia, and new postoperative neurologic deficits.

As an outcome measurement, we investigated death and recurrence dates to analyze mortality and recurrence rates. We also assessed whether the initiation of CTx was delayed due to surgical treatment by comparing the time from surgery to the start of the first CTx, known as Time to CTx (TTC). Response to the initial CTx was evaluated immediately after using magnetic resonance imaging (MRI), categorized as complete response (CR), CR-unconfirmed, partial response (PR), or progressive disease (PD) [11]. Furthermore, we investigated the time to remission (TTR), defined as the time from the surgery to first imaging confirming CR.

### 2.2. Therapeutic Modality

In our institution, biopsy was the standard approach when PCNSL was suspected, even in cases with a solitary and resectable lesion. Surgical intervention was pursued in situations where the diagnosis of lymphoma remained unclear based on preoperative imaging or frozen biopsy or in the presence of acute neurologic deterioration, such as a decline in consciousness owing to elevated intracranial pressure. All patients underwent induction CTx postoperatively. This study investigated the induction and consolidation of CTx types, the implementation of salvage radiotherapy (RTx), and whether salvage whole-brain radiotherapy (WBRT) was performed. The induction CTx was categorized into three groups: high-dose methotrexate (HD-MTX) alone, HD-MTX-based polychemotherapy, and other CTx. This classification was based on current recommendations favoring HD-MTX-based polychemotherapy for the induction CTx of PCNSL [12,13]. The administered dose of WBRT varied between 18 and 50.6 Gy, with a median value of 37 Gy. The median number of fractions was 18, ranging from 19 to 27. Each fraction had a median dose of 2.0 Gy, ranging from 1.8 to 2.0 Gy. Currently, the treatment guidelines indicate no therapeutic superiority among the types of consolidation CTx [5,14]. In this study, patients in the high-risk group underwent consolidation with one of the following options: autologous stem cell transplantation, etoposide, or cytarabine.

### 2.3. Statistical Analysis

All statistical analyses were performed using SPSS version 26.0 (SPSS Inc., Chicago, IL, USA) and R (Version 4.3.1). Categorical data were compared using Fisher’s exact test. Continuous variables were assessed using Student’s t-test or the Mann–Whitney U-test. Additionally, OS was calculated from the time of initial diagnosis to the time of death or censoring, whereas PFS was calculated from the time of first diagnosis to that of relapse or censoring. Survival curves were estimated using the Kaplan–Meier method and compared using the log-rank test. Cox proportional hazards models were employed to evaluate factors associated with OS and PFS. Variables with a *p*-value < 0.5 in univariate analysis were included in the multivariate model. Model fit was assessed using the Likelihood Ratio Test, which demonstrated a significant improvement over the null model for both PFS (−2 Log Likelihood = 200.9; *p* = 0.003) and OS (−2 Log Likelihood = 86.9; *p* = 0.008), supporting the validity of the Cox proportional hazards model for this analysis. Statistical significance was set at *p* < 0.05.

## 3. Results

### 3.1. Patient and Tumor Characteristics

**Figure 1:**
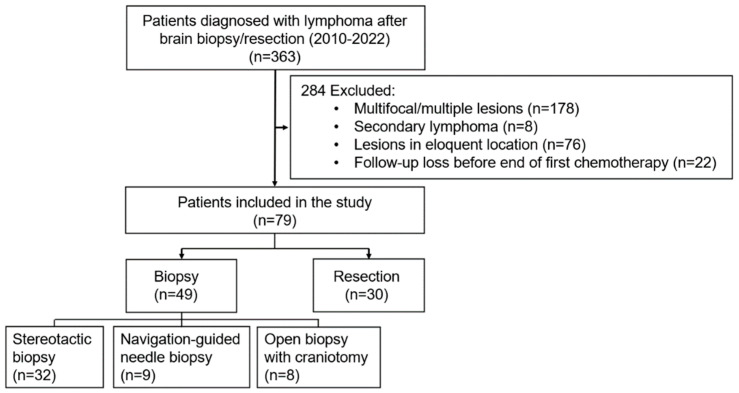
Flowchart illustrating the patient selection process and classification based on the surgical approach.

During the study period, 363 patients were diagnosed with intracranial lymphoma. The study cohort comprised 79 participants, excluding 178 individuals with multifocal or multiple lesions, 8 with secondary lymphoma, 76 with lesions in deep locations, and 22 lost to follow-up before completing the initial CTx cycle. Among them, 49 underwent biopsy and 30 underwent resection. In the biopsy group, 32 individuals had stereotactic biopsies, 9 had navigation-guided needle biopsies, and 8 had open biopsies with craniotomy (Figure 1).

The mean age of the participants was 61.0 ± 12.9 years. No significant differences in sex, age, or underlying comorbidities were observed between the biopsy and resection groups. Comorbidities were categorized into hypertension, diabetes, cardiovascular disease, and others, including chronic conditions such as asthma and chronic kidney disease. Immunocompromised patients were not present in the study cohort. The IELSG score, clinically stratified into low risk (0, 1 points), moderate risk (2, 3 points), and high risk (4, 5 points), showed no significant differences between the two groups. The frontal lobe was the most common location, accounting for 13 cases (26.5%) in the biopsy group and 14 cases (46.7%) in the resection group. The median tumor diameter showed no significant difference between the biopsy group (3.5 cm, range: 1.1–6.7 cm) and the resection group (3.6 cm, range: 1.3–6.6 cm), and no disparities in peritumoral edema were observed. Midline shifting was more pronounced in the resection group, with 10 cases (33.3%), compared to 6 cases (12.2%) in the biopsy group. Additionally, the use of preoperative steroids was significantly higher in the resection group than in the biopsy group (Table 1).

### 3.2. Surgical Outcomes and Complications

**Figure 2:**
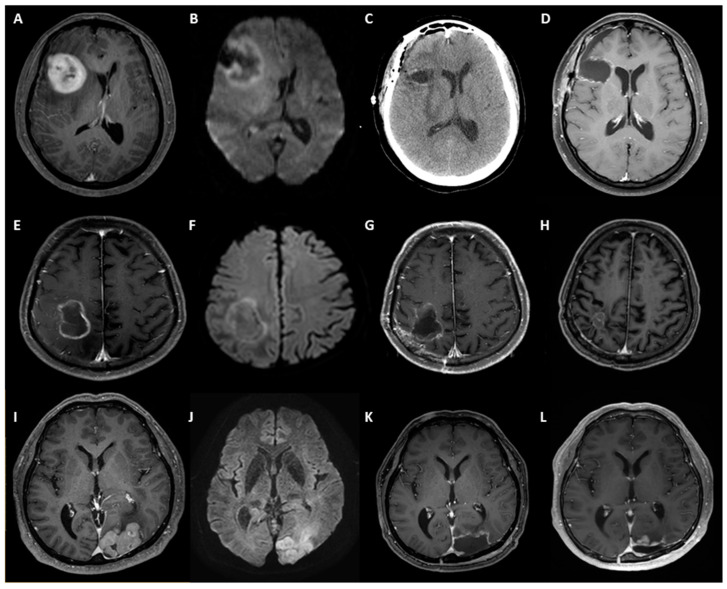
Different appearances of PCNSL in resected cases. One case with midline shifting presented with acute mental deterioration (**A**–**D**). (**A**) Preoperative T1 post-contrast image with clinical suspicion of PCNSL with significant midline shifting and neurological deterioration. (**B**) Diffusion-weighted image (DWI) of the same patient. (**C**) The immediate postoperative CT scan shows improvement in midline shifting. (**D**) T1 post-contrast image at 3 months after resection. Another case underwent resection owing to diagnostic ambiguity (**E**–**H**). (**E**) Preoperative T1 post-contrast image with clinical suspicion of high-grade glioma rather than lymphoma. (**F**) The DWI of the same patient shows mild diffusion restriction. (**G**) Immediate postoperative MRI after GTR. The frozen biopsy revealed high-grade malignancy without a definite identification of cell type. (**H**) T1 post-contrast image at 6 years after resection shows stable disease with CR. A case demonstrating an aggressive course following resection (**I**–**L**). Differential diagnosis from other malignancies, including extra-axial tumors, was needed based on preoperative T1 post-contrast image (**I**) and DWI (**J**). (**K**) GTR on the postoperative T1 post-contrast image. (**L**) A T1 post-contrast image was performed after the completion of the first induction CTx, which was 3 months after resection, indicating PD.

Among the 30 individuals who underwent resection, 10 experienced acute neurological deterioration. Of these, two patients exhibited a decline from alert to drowsy mental status, while eight patients presented with mild hemiparesis. Although biopsy with mannitol administration were viable options for symptom improvement, we opted for surgery for quicker symptom relief which could prevent further neurologic deficit. In two cases, surgery was necessitated by post-biopsy bleeding, and in three cases, total resection followed an open craniotomy owing to the small size of the lesion. Fifteen cases involved frozen biopsy and preoperative radiologic findings that could not conclusively exclude other high-grade malignancies. Figure 2 depicts three distinct surgical scenarios: one involving midline shifting and the other two entailing total resection owing to diagnostic ambiguity.

There were no differences between the resection and biopsy group’s preoperative KPS scores and initial presenting symptoms. Notably, many in the resection group showed symptom improvement within 3 days post-resection or -biopsy (*p* < 0.001). In the resection group, 24 individuals (80%) experienced symptom improvement, 6 (20%) showed no change, and none exhibited symptom deterioration. In contrast, the biopsy group had 10 individuals (20.4%) with symptom improvement, 30 (61.2%) with no change, and 9 (18.4%) with worsened symptoms. Regarding postoperative complications, including surgical site infection, operative site bleeding, postoperative neurologic deficit, and delayed wound healing requiring wound revision, no significant differences were observed between the two groups. Surgical site infection did not occur in either group. Delayed wound healing was observed in one individual in the biopsy group, and postoperative neurologic deficit did not occur in either group. Two patients experienced biopsy site bleeding after the initial biopsy, leading to craniotomy and total resection. These cases were later classified as resection cases in the subsequent prognostic analysis (Table 2).

### 3.3. Clinical Course After Diagnosis and Survival Analysis

Following PCNSL diagnosis, treatment primarily consisted of HD-MTX-based CTx. Two regimens were used: the methotrexate, procarbazine, vincristine regimen and the rituximab, methotrexate, procarbazine, vincristine regimen. Six patients received other CTx: three underwent ifosfamide, carboplatin, etoposide, and dexamethasone following one cycle of MTX, due to poor tolerance; two underwent autologous stem cell transplantation; and one received rituximab, cyclophosphamide, doxorubicin, vincristine, and prednisolone. Nonetheless, there was no statistically significant difference in the types of induction CTx between the two groups. Furthermore, the application of consolidation CTx varied individually. Given the lack of evidence regarding the therapeutic efficacy of consolidation CTx in guidelines, a comparison between the two groups focused solely on its implementation, revealing no significant intergroup difference. In cases of lesion recurrence, the decision to undergo salvage RTx and WBRT showed no disparity between the two groups (Table 1).

Table 3 summarizes additional treatment outcomes, with a mean follow-up period of 41.9 months (range: 3–142). In terms of mortality, the resection group exhibited a 12.0% death rate (6 out of 49), compared to a 20.0% death rate in the biopsy group (6 out of 30). Recurrence rates were 34.1% in the resection group (27 out of 49) and 36.7% in the biopsy group (11 out of 30). The mean TTC for the entire cohort was 14.7 days, with no significant difference observed between the resection group (16.7 days) and the biopsy group (13.5 days) (*p* = 0.189). Significant differences emerged in the response to the first induction CTx between the two groups, with the resection group showing a 73.3% CR rate (22 out of 30) compared to 44.9% in the biopsy group (22 out of 49) (*p* = 0.001). Notably, despite all cases in the resection group undergoing GTR and achieving a CR diagnosis on immediate postoperative imaging, 16.7% (five cases) experienced PD after the first induction CTx. Figure 2I–L depicts the clinical course of one of these cases.

**Figure 3:**
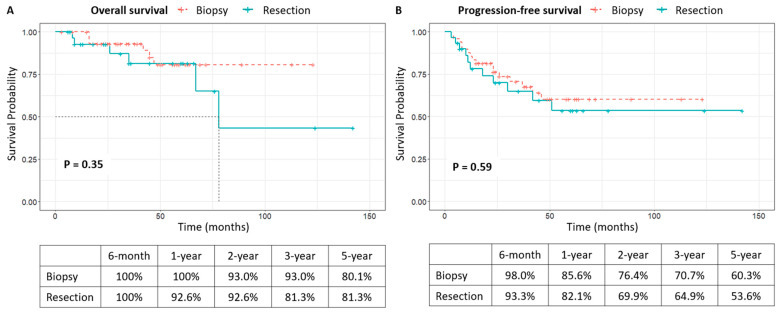
Survival analysis of the patients who underwent biopsy or resection. There was no significant difference between the groups (*p* = 0.35 for overall survival, *p* = 0.59 for progression-free survival). The 5-year overall survival rates were 80.1% for the biopsy group and 81.3% for the resection group, while the 5-year progression-free survival rates were 60.3% and 53.6%, respectively.

Figure 3 shows no significant difference in OS and PFS between the resection and biopsy groups. Mean OS and PFS were 42.1 months and 37.4 months, respectively. The 5-year OS rates were 81.3% for resection and 80.1% for biopsy, while the 5-year PFS rates were 53.6% and 60.3%, respectively. Univariate analysis suggested associations between advanced age (≥65 years) and lack of consolidation CTx with poorer OS, but not statistically significant in multivariate analysis. Similarly, poorer PFS was observed in cases of age ≥ 65 years, no induction CTx, and no consolidation CTx, though not statistically significant in multivariate analysis. The surgical resection did not exhibit statistically significant results in the multivariate analysis (Table 4).

## 4. Discussion

Our study indicates that resection does not improve OS or PFS in patients with solitary, resectable PCNSL. This finding aligns with the prevailing literature and international guidelines, which advocate for biopsy followed by HD-MTX-based CTx as the standard treatment [15,16,17,18]. While complete radiological CR was achieved immediately after GTR, it did not translate into survival benefits. This suggests that radiological remission in PCNSL, unlike in high-grade gliomas, does not necessarily correlate with improved long-term outcomes [19].

Despite the lack of survival benefits, our study highlights potential advantages of resection in specific clinical contexts. Patients in the resection group experienced significant and rapid improvement in neurological symptoms, particularly in cases of acute intracranial pressure elevation or mass effect. This suggests that resection may serve as an effective strategy for symptom relief in carefully selected patients. Importantly, our study did not observe a significant increase in surgical complications in the resection group, consistently with recent findings that resection is a safe modality when performed on appropriately selected patients [20,21]. However, these benefits are limited to symptom management and do not extend to improvements in OS or PFS. Nevertheless, given that resection does not adversely affect long-term survival outcomes and provides rapid neurological improvement, it could be considered more proactively in patients with significant mass effect or those at risk of inconclusive histological diagnosis due to prior steroid use. This approach may enhance diagnostic accuracy and improve immediate clinical outcomes without compromising overall prognosis. Additionally, early postoperative recovery following resection may lead to a more favorable performance status, potentially enabling patients to tolerate and initiate chemotherapy sooner, ultimately optimizing treatment effectiveness and long-term outcomes.

The postoperative neurological outcomes further support the role of resection in symptom management. Patients who underwent resection showed earlier neurological recovery compared to the biopsy-only group, potentially contributing to a more favorable performance status in the early postoperative period. While this could theoretically allow for the more timely initiation of chemotherapy, our study did not find a significant difference in the interval to chemotherapy initiation between the two groups. Nonetheless, the potential impact of early neurological recovery on long-term functional outcomes and quality of life warrants further investigation.

Tumor location may also influence the utility of resection. Although our study focused exclusively on solitary, resectable PCNSL, lesion proximity to eloquent areas was a limiting factor in some cases. Future studies should further explore whether resection offers differential benefits based on tumor location, particularly in cases where mass effect or hydrocephalus is prominent.

Another key consideration is the variability in postoperative adjuvant chemotherapy and salvage treatments between groups. Differences in chemotherapy regimens may have influenced survival outcomes, complicating the isolation of the effects of resection alone. While our study is limited by this heterogeneity, a sensitivity analysis adjusting for chemotherapy variations could provide more insight in future investigations. Given the evolving landscape of PCNSL treatment, including novel agents and immunotherapeutic approaches, prospective studies with standardized chemotherapy protocols would help clarify the interaction between surgical cytoreduction and systemic therapy.

The interpretation of these findings is complicated by the methodological limitations inherent in evaluating the role of resection in PCNSL. One major limitation is the potential selection bias introduced by the retrospective nature of this study and the relatively high rate of exclusions, which could have influenced the observed outcomes. The retrospective nature of our study introduces potential biases, particularly in the selection of patients for resection. Patients in the resection group were more likely to have preoperative steroid use and midline shift, which may indicate a selection bias where patients with more severe presentations were preferentially chosen for resection. This suggests that the absence of a survival benefit in the resection group does not necessarily mean that resection lacks oncological efficacy but rather that these patients may have had a worse baseline prognosis if they had only undergone biopsy. Additionally, variability in postoperative adjuvant CTx and salvage treatments between groups likely confounds the results, making it challenging to isolate the effects of resection alone. Prognosis is influenced by a variety of factors, including immune status, age, performance status, and lesion characteristics, which are difficult to control comprehensively in retrospective analyses. Our study focused on solitary, resectable lesions and controlled for key prognostic factors such as preoperative performance and IELSG score. However, the small sample size and single-center nature of our cohort limit the generalizability of our findings. To address these issues, future studies should adopt prospective designs with standardized treatment protocols and utilize propensity score matching to reduce bias and improve the robustness of comparisons.

Previous studies on the role of resection in PCNSL have produced conflicting results. While many suggest that surgical intervention offers no survival advantage [16,17,18], others have reported potential benefits [22,23,24,25,26,27,28], particularly for PFS [29]. For instance, a large randomized phase III trial conducted by the German PCNSL Study Group in 2012 revealed that patients who underwent biopsy had significantly shorter PFS and OS compared to those who underwent subtotal resection or GTR [30]. However, these benefits diminished when adjusted for factors such as lesion multiplicity, highlighting the complexities of interpreting these findings.

In conclusion, our study reaffirms that total resection does not confer survival benefits in PCNSL. However, it underscores that resection may offer immediate clinical improvements in specific scenarios, such as symptomatic management in patients with significant mass effect. These findings indicate that resection should not be routinely advocated but could be considered as a complementary option in carefully selected cases. Future prospective studies with rigorous designs are needed to clarify the role of resection and establish robust criteria for its use in PCNSL. By addressing these knowledge gaps, we can contribute to more informed and tailored clinical decision-making in this challenging disease.

## 5. Conclusions

Our study emphasizes that GTR does not provide survival benefits in terms of OS or PFS for patients with solitary, resectable PCNSL, reaffirming biopsy followed by HD-MTX-based CTx as the standard treatment. While resection may offer limited symptomatic relief in selective cases, its role remains marginal and should not be routinely advocated. Further prospective studies are needed to better define its utility and refine treatment strategies for PCNSL.

## Figures and Tables

**Table 1:** Patient characteristics and treatment courses.

Characteristics	Biopsy (N = 49)	Resection (N = 30)	*p*-Value
Sex, *n* (%)			0.087
Male	29 (59.2)	11 (36.7)	
Female	20 (40.8)	19 (63.3)	
Age, years, *n* (%)			0.586
≥60	24 (49.0)	12 (40.0)	
<60	25 (51.0)	18 (60.0)	
Comorbidities, *n* (%)			
Hypertension	18 (36.7)	9 (30.0)	0.713
Diabetes	9 (18.4)	4 (13.3)	0.785
Cardiovascular disease	5 (10.2)	0 (0.0)	0.183
Others	5 (10.2)	2 (6.7)	0.897
IELSG score, *n* (%)			0.491
0–1	15 (30.6)	13 (43.3)	
2–3	31 (63.3)	15 (50.0)	
4–5	3 (6.1)	2 (6.7)	
Location, *n* (%)			0.188
Frontal	13 (26.5)	14 (46.7)	
Temporal	7 (14.3)	1 (3.3)	
Parietal	10 (20.4)	7 (23.3)	
Occipital	5 (10.2)	4 (13.3)	
Cerebellum	11 (22.4)	2 (6.7)	
Periventricular or suprasellar	3 (6.1)	2 (6.7)	
Tumor diameter, cm, (range)	3.5 (1.1–6.7)	3.6 (1.3–6.6)	0.754
Peritumoral edema, *n* (%)			0.491
Minimal	1 (2.0)	0 (0.0)	
Moderate	6 (12.2)	6 (20.0)	
Severe	42 (85.7)	24 (80.0)	
Midline shifting, *n* (%)			0.048 *
No	43 (87.8)	20 (66.7)	
Yes	6 (12.2)	10 (33.3)	
Preoperative steroid use			<0.001 *
No	45 (91.8)	10 (33.3)	
Yes	4 (8.2)	20 (66.7)	
Induction CTx			0.437
MTX alone	16 (32.7)	13 (43.3)	
MTX + Other chemotherapeutic agents	30 (61.2)	14 (46.7)	
Others	3 (6.1)	3 (10.0)	
Consolidation CTx			1.000
None	19 (38.8)	12 (40.0)	
Yes	30 (61.2)	18 (60.0)	
Salvage RTx			1.000
None	45 (91.8)	28 (93.3)	
Yes	4 (8.2)	2 (6.7)	
Salvage WBRT			1.000
None	45 (91.8)	27 (90.0)	
Yes	4 (8.2)	3 (10.0)	

* *p*-value < 0.05. CTx: chemotherapy; IELSG: International Extranodal Lymphoma Study Group; MTX: methotrexate; RTx: radiotherapy; WBRT: whole-brain radiotherapy.

**Table 2:** Summary of preoperative and postoperative performance, symptom changes, and complications.

Characteristics	Biopsy (N = 49)	Resection (N = 30)	*p*-Value
KPS (Preop)			1.000
≥80	37 (75.5)	23 (76.7)	
<80	12 (24.5)	7 (23.3)	
KPS (Postop, 1 month)			0.503
≥80	42 (85.7)	28 (93.3)	
<80	7 (14.3)	2 (6.7)	
Initial symptoms, *n* (%)			
Cognition decline	10 (20.4)	7 (23.3)	0.980
Headache	17 (34.7)	6 (20.0)	0.254
Nausea/vomiting	5 (10.2)	3 (10.0)	1.000
Dysarthria	10 (20.8)	5 (16.7)	0.874
Hemiparesis	9 (18.4)	11 (36.7)	0.121
Dizziness	9 (18.4)	5 (16.7)	1.000
Visual disturbance	7 (14.3)	2 (6.7)	0.503
Others	3 (6.1)	0 (0)	0.438
Symptom improvement (Postop, 3 days)			<0.001 *
No change	30 (61.2)	6 (20.0)	
Improved	10 (20.4)	24 (80.0)	
Deteriorated	9 (18.4)	0 (0)	
Postop complications			0.496
Any complication	5 (10.2)	1 (3.3)	
Surgical site bleeding	2 (4.1)	0 (0)	
Delayed wound healing	1 (2.0)	0 (0)	
Systemic complication	2 (4.1)	1 (3.3)	

* *p*-value < 0.05. KPS: Karnofsky performance status; Preop: preoperative; Postop: postoperative.

**Table 3:** Treatment outcomes of the patients.

Characteristics	Total (N = 79)	Biopsy (N = 49)	Resection (N = 30)	*p*-Value
Follow-up, months				0.904
mean (range)	41.9 (3–142)	41.5 (3–123)	42.5 (6–142)	
Death, *n* (%)	12 (15.2)	6 (12.0)	6 (20.0)	0.542
Recurrence, *n* (%)	27 (34.1)	16 (32.7)	11 (36.7)	0.904
TTC, days				0.189
Mean (range)	14.7 (1–47)	13.5 (1–47)	16.7 (1–43)	
TTR, months				0.018 *
Mean (range)	4.4 (1–20)	4.9 (2–20)	3.5 (1–8)	
Response to first induction CTx				0.001 *
CR	44 (55.7)	22 (44.9)	22 (73.3)	
CRu/PR	27 (34.2)	24 (49.0)	3 (10.0)	
PD	8 (10.1)	3 (6.1)	5 (16.7)	

* *p*-value < 0.05. CR: complete response; CRu: complete response unconfirmed; PR: partial response; PD: progressive disease; CTx: chemotherapy; TTC: time to chemotherapy; TTR: time to remission.

**Table 4:** Treatment outcomes of univariate and multivariate analyses of OS and PFS.

	No. of Deaths/Total No. of Patients (%)	Univariate		Multivariate			No. of Recurrences /Total No. of Patients (%)	Univariate		Multivariate		
		At 5 Years	Log-Rank	HR	95% CI	*p* Value		At 5 Years	Log-Rank	HR	95% CI	*p* Value
Overall	12/79 (15.2)						27/79 (34.2)					
Sex			0.567						0.144			
Female	6/40 (15.0)	83.5 ± 6.9					11/40 (27.5)	67.2 ± 8.4				
Male	6/39 (15.4)	78.4 ± 9.5					16/39 (41.0)	46.5 ± 9.9		1.458	0.658–3.227	0.353
Age (years)			0.005 *						0.007 *			
<65	4/43 (9.3)	92.7 ± 4.1					11/43 (25.6)	70.5 ± 7.7				
≥65	8/36 (22.2)	46.4 ± 16.0		2.076	0.421–10.228	0.369	16/36 (44.4)	34.6 ± 11.6		1.889	0.732–4.870	0.188
Tumor size (mm)			0.913						0.578			
<30	5/31 (16.1)	83.3 ± 8.0					12/31 (38.7)	58.5 ± 9.3				
≥30	7/48 (14.6)	79.7 ± 7.7					15/48 (31.3)	56.8 ± 9.0				
IELSG score			0.479						0.904			
0–1	3/29 (10.3)	83.8 ± 8.8					9/29 (31.0)	64.8 ± 9.8				
2–3	9/45 (20.0)	78.4 ± 7.6					17/45 (37.8)	53.4 ± 8.7				
4–5	0/5 (0.0)	0.0					1/5 (20.0)	66.7 ± 27.2				
Surgery			0.351						0.586			
Biopsy	6/49 (12.2)	80.7 ± 7.4					16/49 (32.7)	60.3 ± 8.2				
Resection	6/30 (20.0)	81.3 ± 8.7		1.900	0.585–6.175	0.286	11/30 (36.7)	53.6 ±10.8				
Induction CTx			0.151						0.015 *			
MTX based CTx	10/73 (13.7)	83.7 ± 5.5					22/73 (30.1)	63.4 ± 6.5				
Others	2/6 (33.3)	55.6 ± 24.8		2.503	0.495–12.650	0.267	5/6 (83.3)	22.2 ± 19.2		2.172	0.782–6.034	0.137
Consolidation CTx			0.001 *						0.002 *			
None	13/22 (59.1)	52.2 ± 13.7					16/31 (51.6)	29.6 ± 10.5				
Yes	5/27 (18.5)	95.0 ± 3.5		0.237	0.044–1.289	0.096	11/48 (22.9)	73.0 ± 7.3		0.509	0.198–1.306	0.160
Salvage RTx			0.372									
None	10/73 (13.7)	80.5 ± 6.2										
Yes	2/6 (33.3)	83.3 ± 15.2		3.499	0.638–19.183	0.149						

* *p*-value < 0.05. CI: confidence interval; CTx: chemotherapy; HR: hazards ratio; IELSG: International Extranodal Lymphoma Study Group; MTX: methotrexate; OS: overall survival; PFS: progression-free survival; RTx: radiotherapy; SE: standard error.

## Data Availability

The datasets generated during the current study are available from the corresponding author upon reasonable request.

## Abbreviations

The following abbreviations are used in this manuscript:

CNS	Central nervous system
CR	Complete response
CTx	Chemotherapy
GTR	Gross total resection
HD-MTX	High-dose methotrexate
MRI	Magnetic resonance imaging
MPV	Methotrexate, procarbazine, vincristine
OS	Overall survival
PCNSL	Primary central nervous system lymphoma
PD	Progressive disease
PFS	Progression-free survival
R-MPV	Rituximab, methotrexate, procarbazine, vincristine
RTx	Radiotherapy
STR	Subtotal resection
TTC	Time to chemotherapy
TTR	Time to remission
WBRT	Whole-brain radiotherapy
